# Attenuated expression of MTR in both prenatally androgenized mice and women with the hyperandrogenic phenotype of PCOS

**DOI:** 10.1371/journal.pone.0187427

**Published:** 2017-12-12

**Authors:** Lei Lei, Lijun Ding, Jing Su, Mengyuan Liu, Qingqing Shi, Jianjun Zhou, Haixiang Sun, Guijun Yan

**Affiliations:** 1 Center for Reproductive Medicine, Drum Tower Clinic Medical College of Nanjing Medical University, Nanjing, Jiangsu, China; 2 Department of Obstetrics and Gynecology, Nanjing First Hospital, Nanjing Medical University, Nanjing, Jiangsu, China; 3 Center for Reproductive Medicine, Department of Obstetrics and Gynecology, the Affiliated Drum Tower Hospital of Nanjing University Medical School, Nanjing, Jiangsu, China; China Agricultural University, CHINA

## Abstract

Polycystic ovary syndrome (PCOS) is a common endocrine, metabolic and heterogeneous disorder in women of reproductive age, the exact etiology of which remains unknown. To unravel the molecular mechanisms underlying the hyperandrogenic phenotype of PCOS, prenatally androgenized (PNA) mice were used to mimic this phenotype in women with PCOS. Using microarray analysis, 1188 differentially expressed genes, including 671 upregulated and 517 downregulated genes, were identified in ovaries from PNA mice. Five differentially expressed genes (*Aldh1a7*, *Bhmt*, *Mtr*, *Nrcam*, *Ptprg*) were validated, and decreased MTR expression was shown in ovaries of PNA mice. In addition, results from qRT-PCR showed decreased *MTR* expression in granulosa cells (GCs) from women with the hyperandrogenic phenotype of PCOS. Serum levels of S-adenosyl methionine (SAM), the downstream product of MTR, were also decreased in PNA mice and women with the hyperandrogenic phenotype of PCOS. Our study provides evidence that the hyperandrogenic phenotype of PCOS is linked to abnormal folate one-carbon metabolism.

## Introduction

Polycystic ovary syndrome (PCOS) is one of the most common endocrine and metabolic disorders, affecting about 5%-15% of women of reproductive age [1]. Symptoms of PCOS include amenorrhea or oligomenorrhea, hyperandrogenism, and polycystic ovarian morphology. As a heterogeneous disorder, PCOS shows evidence of a genetic predisposition among patients, but the exact etiology remains unknown [2].

Previous studies have been conducted on many candidate genes for PCOS, principally related to reproductive hormones, insulin resistance, and chronic inflammation, including follicle-stimulating hormone receptor (*FSHR*) [3], insulin receptor (*INSR*) [4], and tumor necrosis factor (*TNF*) [5]. Microarraywas also used to screen for differentially expressed genes in PCOS using ovaries [6], theca cells [7], oocytes [8], cumulus cells [9], and even skeletal muscle [10] and adipose tissue [11]. The first genome-wide association studies (GWAS) and subsequent follow-ups were performed in Han Chinese women, and investigators identified the following PCOS candidate loci: *DENND1A*, *INSR*, *YAP1*, *C9orf3*, *RAB5B*, *HMGA2*, *TOX3*, *SUMO1P1/ZNF217*, *THADA*, *FSHR*, and *LHCGR* [12, 13]; *DENND1A* was validated by another study [14].

Unfortunately, susceptibility genes for PCOS were often controversial in nature in previously reported studies. The controversy is partly due to ethnic differences, but different PCOS phenotypes could also be another reason [1]. Animal models may help to investigate the pathophysiologic mechanisms in a certain phenotype of PCOS. As an important feature of PCOS, hyperandrogenism is also one of the diagnostic criteria for this disease, a feature distinct from metabolic dysfunction. Therefore, to investigate the etiology of the hyperandrogenic phenotype of PCOS, a prenatally androgenized (PNA) mouse model was validated and used for microarray analysis. Differentially expressed genes (1188) were identified in ovaries from PNA mice, and five of these (*Aldh1a7*, *Bhmt*, *Mtr*, *Nrcam*, *Ptprg*) were validated by qRT-PCR; MTR expression was then further evaluated by western immunoblotting analysis and immunohistochemistry. MTR is the only mammalian enzyme that metabolizes N^5^-MeTHF to regenerate the active cofactor tetrahydrofolate (THF)[15]. The overall reaction converts 5-methyltetrahydrofolate (N^5^-MeTHF) into THF while transferring a methyl group to homocysteine(Hcy) to form methionine(Met). Met is further used to synthesize S-Adenosyl methionine(SAM), the main methyl donor of DNA methylation, under ATP action. Decreased *MTR* expression in granulosa cells (GCs) from women with the hyperandrogenic phenotype of PCOS was also validated by qRT-PCR. Additionally, serum levels of SAM, the downstream product of MTR, were decreased in both PNA mice and the hyperandrogenic phenotype of women with PCOS. The present study, therefore, provides novel basic information on the relationship between MTR and the hyperandrogenic phenotype of PCOS.

## Materials and methods

### Animals

All experimental procedures were performed in accordance with the guidelines of the Experimental Animals Management Committee (Jiangsu Province, China) and were approved by Nanjing Drum Tower Hospital Experimental Animals Welfare &Ethical committee (20150302).Adult ICR mice (females, 6 weeks of age, n = 50; males, 10 weeks of age, n = 10) were purchased from the Animal Experimental Center of Yangzhou University (Jiangsu Province, China), and housed in the Drum Tower Hospital Animal Experimental Center (Jiangsu Province, China) at 22°C, on a 12 h light/12 h dark cycle with lights on at 07:00 am, and with ad libitum access to chow and water. Females were mated with males and checked for copulatory plugs daily. The date of the plug was considered day 1 of gestation. Pregnant dams were injected daily s.c. with 70 μl of sesame oil containing 350 μg of DHT (521-18-6, Sigma, USA)or sesame oil vehicle on days 16–18 of gestation, and female offspring were studied. The mice were euthanized through anesthesia with chloral hydrate. Tissues and blood were harvested from all animals post euthanization.

### Assessment of estrous cyclicity and fertility

The body weights of PNA and control mice were recorded, starting at 21 days of age. Vaginal smears were obtained daily in all adult mice from 2 months of age for 3 weeks or those showing consecutive estrous cycles. The fertility of adult mice (n = 6 each group) was tested by mating with proven fertile ICR males (1: 1) for 3 months. The numbers of litters and pups per litter were recorded.

### Testosterone and S-adenosyl methionine measurements in mice

The mice were anesthetized with chloral hydrateon diestrus, and blood was collected from the posterior orbital venous plexus. The blood samples were then centrifuged and the serum was frozen at -80°C for hormonal analysis. The concentration of testosterone (T) was measured using an ELISA kit (YANYU, Shanghai, China), and the serum levels of SAM were also detected using an ELISA kit (CEG414Ge, Cloud-Clone Corp, Wuhan, China).

### Ovarian histology and follicle counting

After blood was collected from mice, ovaries were removed, weighted and then fixed in Bouin’s solution. Ovarian index was calculated using ovarian weight * 1000/body weight * 100 (%). After fixation for 4–6 h, the samples were dehydrated and embedded in paraffin, sections cut at 5 μm, and every fifth section was stained with hematoxylin and eosin (H&E). Follicle counting was performed using an unbiased stereologic method. Only follicles containing an oocyte nucleus were counted. The total number of follicles was then multiplied by five. Follicles were classified according to the classification system in a previous report [16].

### Microarray analysis

The microarray was conducted by Genechem Co., Ltd. (Shanghai, China). The GeneChip^®^ Mouse Genome 430 2.0 Array (900496, Affymetrix) was used for the experiment, and 2 groups of samples were tested, with each group containing 5 mouse ovaries. RNA was extracted using Trizol reagents, and qualified with a NanoDrop 2000 and Agilent Bioanalyzer 2100. The quality control standards were as follows:1.7 < A260/A280 < 2.2 (ThermoNanoDrop 2000), RIN ≥ 7.0 and 28S/18S > 0.7 (Agilent 2100 Bioanalyzer). The qualifying samples were entered into the microarray experiment.

### Quantitative real-time PCR (qRT-PCR)

RNA was extracted using Trizol reagents and measured with spectrometry for OD260/280. cDNA was prepared with 5× All-In-One RT MasterMix (G490, ABM, USA) according to the manufacturer’s recommendations. Real-time PCR was performed with a SYBR-Green Mixture (Bio-Rad, USA). Primers were searched from https://pga.mgh.harvard.edu/primerbank/. The sequences of specific PCR primers in this study are listed in S1 Table. Primers were synthesized by Sangon (Sangon Biotech, Shanghai, China).Each sample was run in triplicate as follows: 2 μl cDNA, 1 μl primer, 7 μldd H_2_O, and 10 μl SYBR Green Master Mix in a total volume of 20 μl; and 18s rRNA was measured as an internal control. The PCR procedure was as follows: initial denaturation at 95°Cfor 1 min followed by 40 cycles of amplification (denaturation at 95°C for 15 sec, annealing at 60°C for 1 min), and then primer template extension at 72°C for 3 min. Melting curve analysis was performed to confirm the specificity of amplification, and the relative *MTR* level was determined using 2^−ΔΔCT^.

### Immunohistochemistry (IHC)

The expression of MTR was also confirmed by immunohistochemistry. Sections were baked at 65°C, dehydrated, and incubated in 3% H_2_O_2_. Sections were subjected to antigen retrieval by boiling in 10 mM citrate buffer (pH 6.0), and rinsed in TBS. We added solution A (Biotin block), washed with TBS, added solution B (Biotin block), and we again washed sections with TBS. Sections were incubated in normal goat serum for 1 h at 4°C, incubated with anti-MTR (1:200, ab66039, Abcam, UK) in 3% BSA overnight at 4°C, incubated in biotinylated goat anti-rabbit IgG (1:200 dilution) for 2 h at room temperature, and then washed with PBS. We added reagent SABC (12E02A, BOSTER, Wuhan, China) and incubated sections at 37°C for 20 min, washed with PBST, visualized binding with DAB, and terminated the incubation with distilled water. Slides were stained with hematoxylin, dehydrated, and mounted. The average integrated optical density (IOD) was measured for each sample by Image-pro Plus 6.0 (Media Cybernetics, USA).

### Western blot(WB) analysis

Ovarian lysates from mice were separated by SDS/PAGE and transferred onto nitrocellulose membranes. Membranes were probed with polyclonal MTR antibodies (1:2000, ab66039, Abcam, UK), and blots were visualized by using peroxidase-conjugated second antibody and an ECL detection kit (Amersham Pharmacia Biosciences). Western blot data were quantified and normalized to GAPDH (1:10000, Bio-Rad, USA).

### Clinical samples

Women with the hyperandrogenic phenotype of PCOS and age-matched controls were recruited from the Center for Reproductive Medicine, Nanjing Drum Tower Hospital, from January to December 2016. All subjects were unrelated Han Chines eand were recruited after providing informed written consent. The study was approved by the Medical Ethical Committee of Nanjing Drum Tower Hospital. The diagnosis of PCOS was based upon the 2003 Rotterdam Diagnostic Criteria [17].All patients with PCOS in this study had oligomenorrhea, hyperandrogenism, and a clear diagnosis of polycystic ovaries by ultrasonography. The controls were fertile women undergoing IUI or IVF for male factor infertility; women with ovarian factor or complications affecting ovulation (e.g., hypothyroidism, diabetes mellitus, endometriosis, hyperprolactinemia) were excluded.

### Hormonal and S-adenosyl methionine measurements in women

On cycle days 2 or 3, venous blood samples were collected; and serum hormone levels, including follicle-stimulating hormone (FSH), luteinizing hormone (LH), prolactin (PRL), estradiol (E_2_) and testosterone (T), were measured using ELISA Kits (FSH, 33520; LH, 33510; PRL, 33530; E_2_, 33540; T, 33560; Beckman Coulter, Inc, USA). Blood samples were also collected and centrifuged on the day before HCG was injected, and serum was frozen at -80°C for SAM analysis (CEG414Ge, Cloud-Clone Corp, Wuhan, China). Ultrasonography was performed on cycle day 4 or 5 to measure the thickness of endometrium, count the number of antral follicles and confirm the absence of corpus luteum.

### Isolation of human granulosa cells

All women were injected with gonadotropin-releasing hormone (GnRH) agonist at the beginning of the midluteal phase, and then ultrasonographic scans and serum estradiol assays were performed to monitor follicular size. When 3 or more follicles with a mean diameter of 16 mm were observed, 5000–10,000 IU human chorionic gonadotropin (HCG) was injected. Ultrasound-guided oocyte retrieval was performed 36 hours later. The granulosa cells (GCs) around oocytes were collected and washed twice with Dulbecco’s modified Eagle’s medium (DMEM) after removal of the oocyte, and kept in TRIzol (15596–018, Invitrogen, USA) at -80°C for RNA isolation.

### Statistical analysis

Statistical analysis was performed using SPSS 19.0 (IBM, USA). The means ± SD of the data were calculated. Either Student’s *t*-test or *t’*-test was used to determine the significance between the two groups. A P value < 0.05 was considered to be statistically significant.

## Results

### Hyperandrogenism disrupts estrous cyclicity, impairs fertility and increases serumtestosterone levels in PNA mice

**Fig 1:**
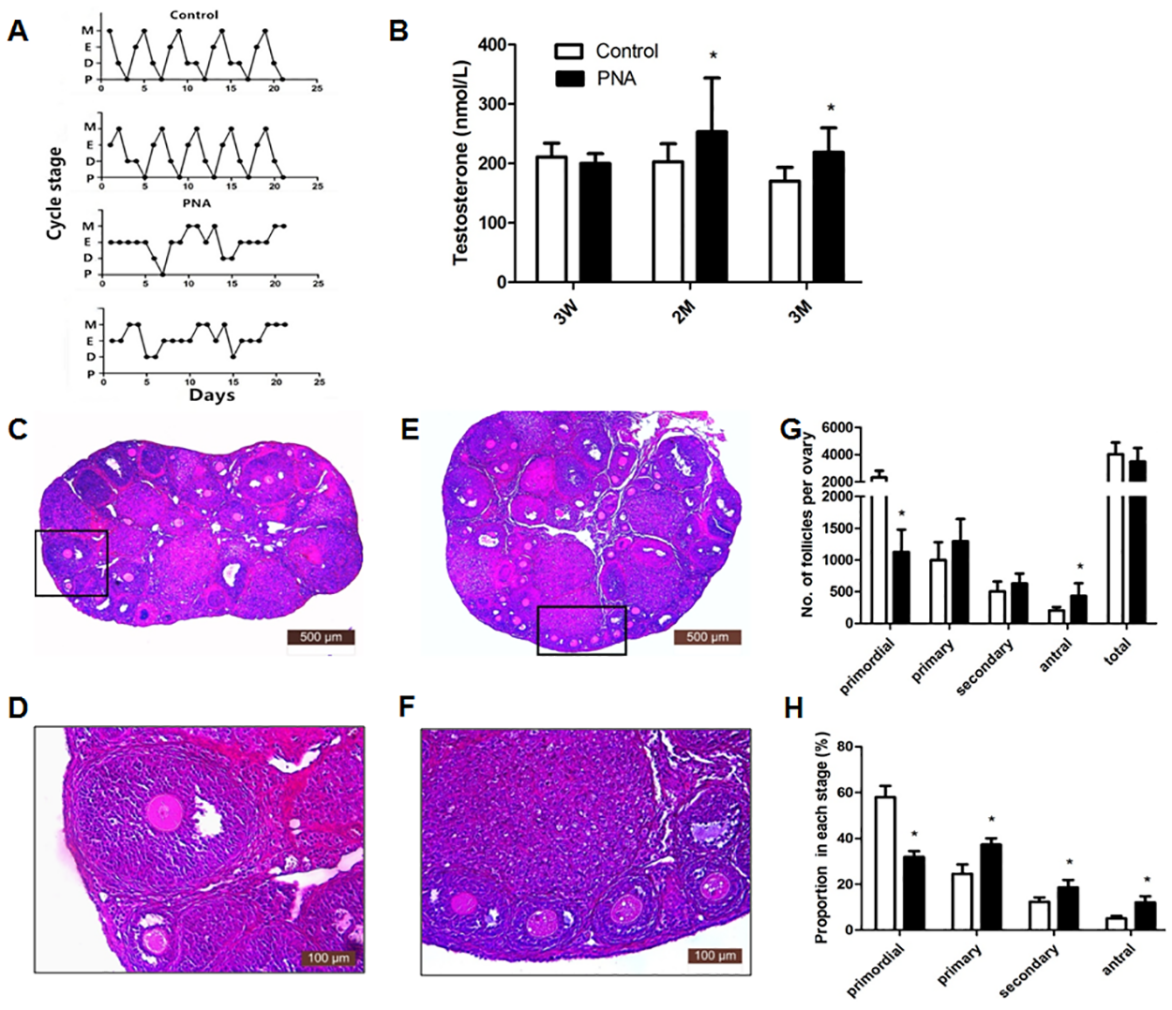
Estrous cycle, serum testosterone levels, ovarian morphology and follicles counting of mouse. (A) Representative estrous cycles in control (*Upper*) and PNA mice (*Lower*) (M, metestrus; E, estrus; D, diestrus; P, proestrus). (B) T levels in diestrus mice. (C) (D) HE stained ovarian section of control mouse at 3 months. (E) (F) HE stained ovarian section of PNA mouse at 3 months. (G) Follicle counting at 3 months, numbers represent total counting of every fifth section from serially sectioned ovaries. (H) Proportion of follicles in each stage.(*: *P*< 0.05).

PNA mice exhibited prolonged estrous cycles (8.79 ± 2.58 days, n = 15) compared with controls (4.5 ± 0.46 days, n = 15) (*P*< 0.001),which was mainly due to the increased durations of estrus and metestrus. An absence of proestrus was also be observed in the PNA mice (Fig 1A, S1A and S1B Fig). Adult PNA mice had fewer litters and smaller litter sizes (1.67 ± 0.52 litters, 4.83 ± 1.25 pups/litter) compared with the controls (3.33 ± 0.52 litters, 14.65 ± 0.91 pups/litter) during a 3- month period (*P*< 0.001) (S1C and S1D Fig).

To examine the endocrine factor contributing to abnormal cyclicity in PNA mice, serumT was measured. T levels at 3 weeks of age showed no difference between the two groups (*P*> 0.05), but PNA mice had increased T levels at 2 months of age (253.14 ± 90.60 nmol/L, n = 21 vs. 202.79 ± 30.32 nmol/L, n = 15; *P* = 0.026) and 3 months of age (218.80 ± 41.05 nmol/L, n = 21 vs.170.24 ± 23.21 nmol/L, n = 13; *P*< 0.001) (Fig 1B). These data suggest that PNA treatment elevated T levels in adult PNA mice, but not before puberty. It was also found that T levels at 3 months decreased compared with levels at 3 weeks in the control group, which was not found in the PNA mice.

### Hyperandrogenism alters ovarian morphology and folliculogenesis in PNA mice

Body weight of PNA mice (13.91 ± 2.84 g, n = 18) was increased compared with the control group (12.01 ± 2.46 g, n = 29) at 3 weeks of age (*P* = 0.020), but there was no difference between the two groups after puberty (S1E Fig). Ovarian weight of the PNA mice (33.36 ± 8.61 ×10^−4^ g, n = 18) was increased compared to the control group (26.45 ± 7.67 ×10^−4^ g, n = 29) (*P* = 0.006), but the ovarian index was not significantly different between the two groups at 3 weeks of age. At 2 months, the ovarian weight and ovarian index of the PNA mice were both significantly decreased compared to the control group (52.03 ± 21.04 ×10^−4^ g, 35.38 ± 10.60%, n = 14 vs.77.67 ± 21.04 ×10^−4^ g, 50.54 ± 16.27%, n = 12; *P* = 0.008, *P* = 0.009). At 3 months of age, there was no statistical difference in ovarian weight or index between the two groups (S1F and S1G Fig).

Compared with the controls, ovaries in the adult PNA mice (3 months old) exhibited a greater number of small antral follicles, fewer corpora lutea, but contained cyst-like structures (Fig 1C–1F). Although the total number of antral follicles was increased, the number of preovulatory follicles was decreased in the ovaries of adult PNA mice. In addition, antral follicles of the PNA mice exhibited a thinner granulosa cell layer.

Follicle counting (n = 10 each group) showed that PNA mice possessed fewer primordial follicles (1126 ± 352.79 vs. 2318.5 ± 491.95, *P*< 0.001) and more antral follicles than controls (433 ± 200.52 vs. 204 ± 55.26, *P* = 0.006). There was no difference in the number of total follicles, or in primary or secondary follicle number (*P*> 0.05) (Fig 1G).However, when we analyzed the proportion of follicles at each stage, the PNA mice exhibited a lower percentage of primordial follicles (31.92 ±2.50% vs. 57.95 ± 4.98%, *P*< 0.001) and a higher percentage of the other three follicular stages (primary, 37.33 ± 2.76% vs. 24.55 ± 4.10%, *P*< 0.001; secondary, 18.65 ± 3.26% vs. 12.389 ± 1.85%, *P*< 0.001; antral,12.10 ± 2.70% vs. 5.12 ± 1.11%,*P*< 0.001) (Fig 1H). These results suggested that follicles were overly activated in the PNA mice.

### Novel differentially expressed genes in PNA mice

**Fig 2:**
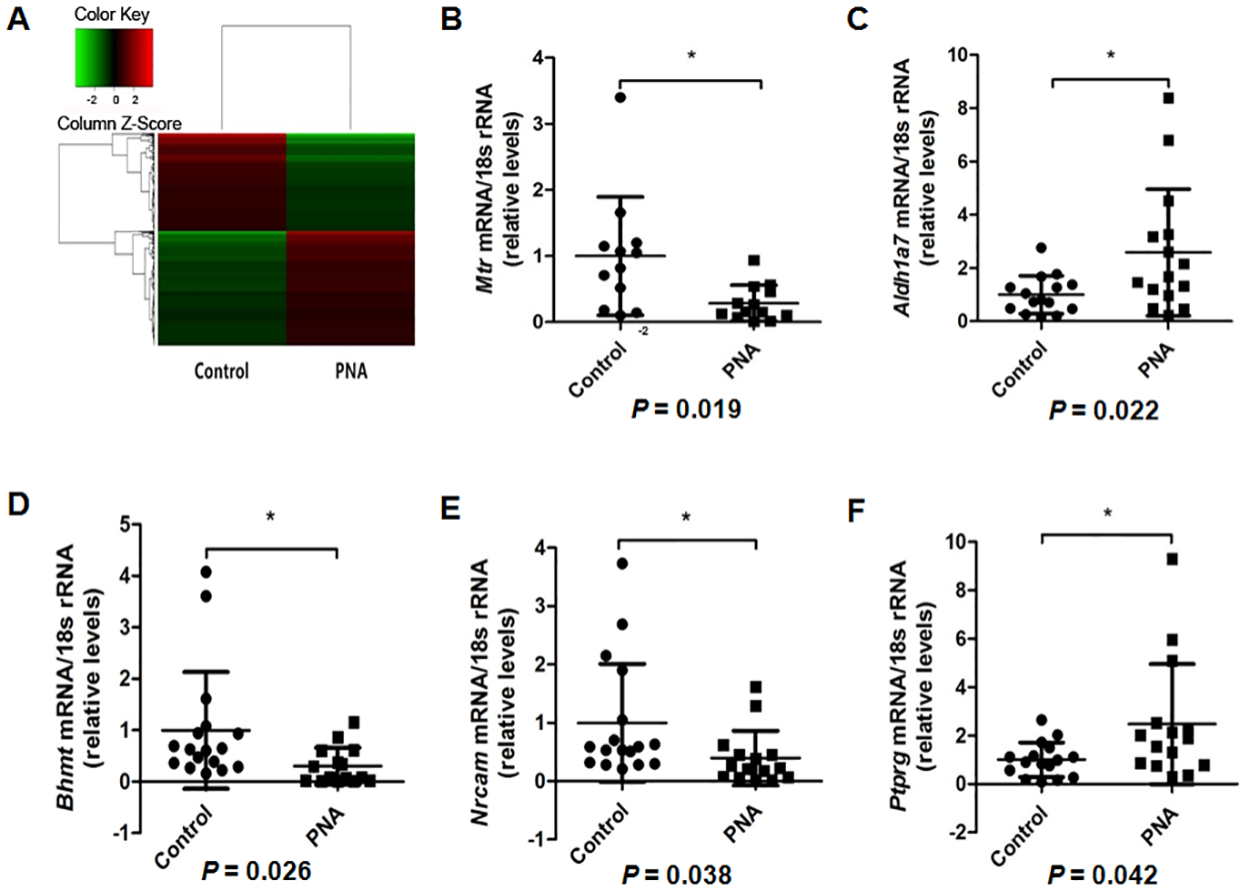
Microarrayanalysis and validation. (A) Heatmap of microarray analysis. (B) -(F) Five differentially expressed genes were validated by qRT-PCR(B, *Mtr*; C, *Aldh1a7*; D, *Bhmt*; E, *Nrcam*; F, *Ptprg*).(*: *P*< 0.05).

RNA extracted from ovaries was qualified using a NanoDrop 2000 and Agilent Bioanalyzer 2100 (S2 Fig). Over 39,000 transcripts were analyzed with the GeneChip^®^ Mouse Genome 430 2.0 Array by using the selection criteria of fold change ≥ 1.5, and a total of 671 gene transcripts were activated while 517 others were repressed in PNA mouse ovaries (Fig 2A). Genes involved in androgen biosynthesis and LH secretion (*Hsd11b1*, *Hsd17b2*, *Hsd17b7*, *Cyp11a1*, *Cyp17a1*, *Cyp19a1*, *Cyp2d22*, *Cyp39a1*, *Cyp4f18* and *Lhcgr*), ovarian function and folliculogenesis (*Pten*, *Amh* and *Nppc*), and inflammation (*CCL2*, *CCL5*, *CCL7*, *CXCL1*, *CXCL9*, *CXCL10*, *and CCR7*) were among those with differential expression.

The differentially expressed genes were enriched and analyzed according to the gene information of all pathways in KEGG and BIOCARTA. The top 3 pathways were cancer, cytokine-cytokine receptor interaction, and chemokine signaling pathway; the top 3 under biological processes were signal transduction, multicellular organismal development and anatomical structure development; and the top 3 molecular functions were receptor binding, receptor activity and enzyme regulator activity (S2–S4 Tables).

To validate the expression changes identified by microarray analysis, the expression levels of 5 differentially expressed genes (*Aldh1a7*, *Bhmt*, *Mtr*, *Nrcam*, *Ptprg*) were assessed by qRT-PCR (Fig 2B–2F). Results showed that the 5 selected genes displayed a trend similar to that of the microarray data, thus confirming the validity of the microarray results.

**Fig 3:**
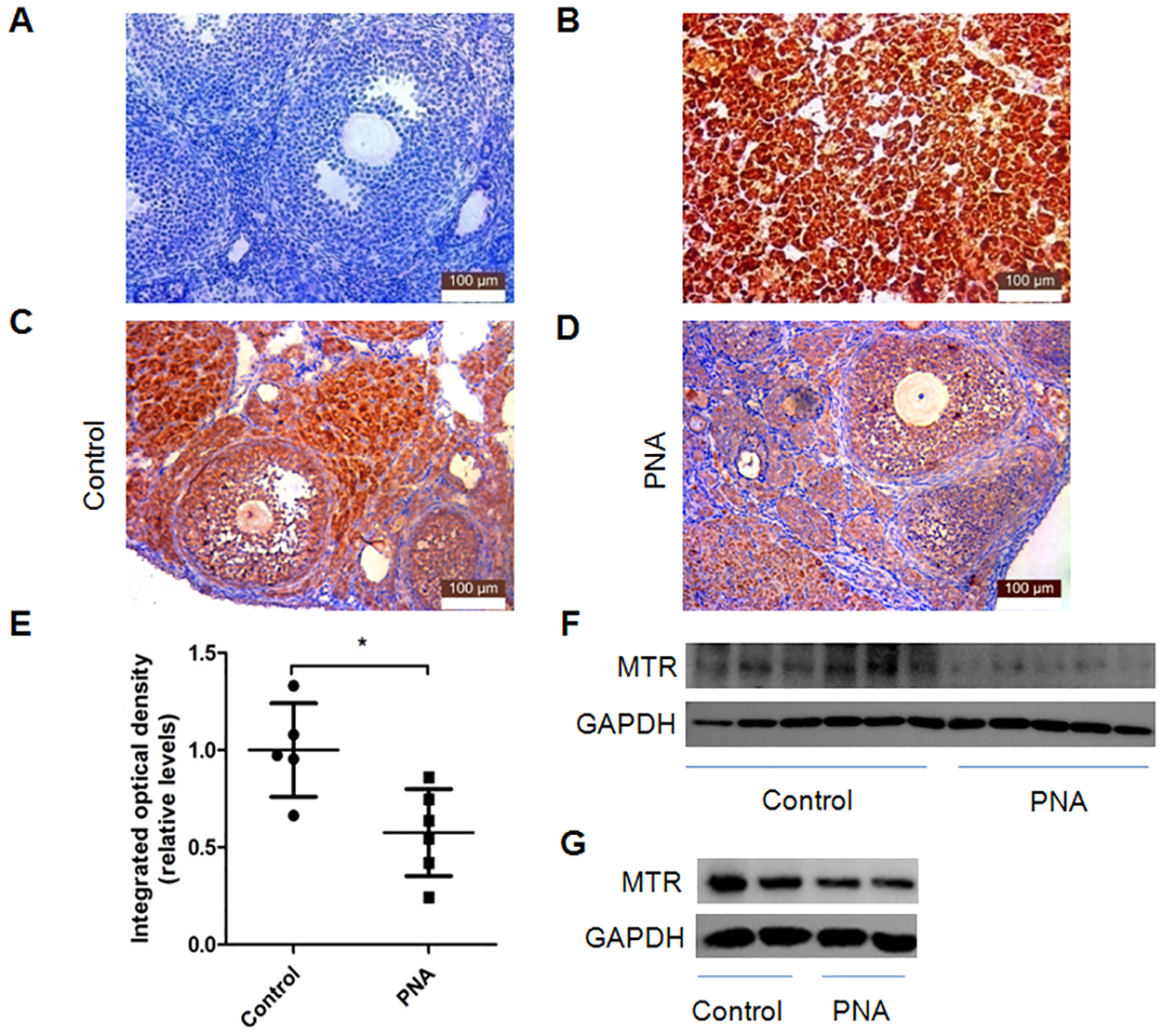
Differentially expressed MTR in mice. (A)–(D) Immunohistochemistry. (A) Negative control (ovary). (B) Positive control (pancreas). (C) Ovary of control mouse. (D) Ovary of PNA mouse. (E) IOD of MTR was significantly lower in PNA mice. (*: *P*< 0.05). (F) WB showed expression of MTR in ovaries of PNA mice decreased at 3 months age. (G) WB showed expression of MTR in ovaries of PNA mice decreased at 3 weeks age.

By IHC, we found that MTR was mainly expressed in granulosa cells, and rarely expressed in theca cells (Fig 3C and 3D).Subcellular localization of MTR showed the greatest expression in the cytoplast and was not appreciably observed in the nucleus (Fig 3B–3D). The expression of MTR in the ovaries of adult PNA mice (at 3 months of age) was significantly decreased compared with the controls (*P* = 0.014; Fig 3E).

Western-blot analysis also confirmed the above results. The expression of MTR in the ovaries of PNA mice was decreased at both 3 weeks and 3 months of age (Fig 3F and 3G). Although we also observed for adult mice in the control group an attenuated expression of MTR compared with mice before puberty, this change was not obvious in PNA mice (S3 Fig).

These results indicated that the expression of MTR was decreased in the ovaries of PNA mice even prior to puberty.

### Hyperandrogenism decreases serum SAM levels in PNA mice

**Fig 4:**
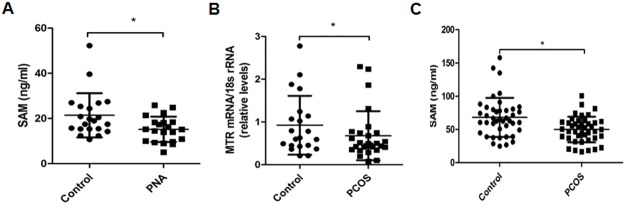
Differentially expressed *MTR* in women, serum SAM levels in mouse and women. (A) PNA decreases serum SAM levels in mouse. (B) Women with PCOS have low- expressed *MTR* in GCs. (C) low- SAM level in serum of women with PCOS.(*: *P*< 0.05).

SAM is the downstream product of MTR, and ELISA showed that serum SAM levels were significantly decreased in PNA mice (21.41 ± 9.79 ng/ml) compared with the control group (15.20 ± 5.62 ng/ml)(n = 21 in each group, *P* = 0.016; Fig 4A).

### Women with PCOS have attenuated expression of *MTR* in GCs and lowered SAM levels in serum

Characteristics of patients are shown in Table 1. The control groups and patients showing the hyperandrogenic phenotype of PCOS were matched for age. Serum E_2_ and PRL were not significantly different between the two groups. However, BMI, Mean arterial pressure (MAP), ovarian volume, serum LH, T and the LH/FSH ratio of the patients with PCOS were higher than the controls (BMI, 23.38 ± 3.87 kg/m^2^ vs. 21.71 ± 3.07 kg/m^2^; MAP, 90.90 ± 11.33mmHg vs. 85.41± 9.01mmHg; ovarian volume, 8.33 ± 3.79 cm^3^ vs. 4.82 ± 2.04cm^3^; LH,10.19 IU/L ± 7.66 vs. 4.28 ± 1.61 IU/L; T, 0.67 ± 0.29 vs. 0.42 ± 0.14; LH/FSH, 1.70 ± 1.18 vs. 0.63 ± 0.23; *P*< 0.05). Additionally, more oocytes were retrieved from PCOS patients (15.19 ± 5.00) compared to controls (12.18 ± 4.19) (*P*< 0.001). These results showed that many physiologic differences were typically observed between PCOS patients and controls.

**Table 1:** Clinical characteristics of PCOS patients and controls.

Characteristic	Control (n = 67)	PCOS (n = 74)	*P* value
**Age (y)**	27.24± 3.07	27.51 ± 2.49	NS
**BMI (kg/m**^**2**^**)**	21.71± 3.07	23.38 ± 3.87	0.005
**mean arterial pressure (mmHg)**	85.41± 9.01	90.90 ± 11.33	0.002
**Ovary volume (cm**^**3**^**)**	4.82±2.04	8.33 ± 3.79	< 0.001
**FSH (IU/L)**	6.81± 1.36	6.01± 1.72	0.003
**LH (IU/L)**	4.28± 1.61	10.19 ± 7.66	< 0.001
**E**_**2**_ **(pg/mL)**	42.45± 19.45	52.91 ± 58.61	NS
**PRL (ng/mL)**	15.77± 6.46	15.00±7.50	NS
**T (ng/mL)**	0.42 ± 0.14	0.67 ± 0.29	< 0.001
**LH/FSH**	0.63± 0.23	1.70 ± 1.18	< 0.001
**Oocytes retrieved**	12.19± 4.20	15.19 ± 5.00	< 0.001

The expression of *MTR* in GCs from women with the hyperandrogenic phenotype of PCOS was significantly decreased compared with controls (*P* = 0.036; Fig 4B). Furthermore, serum SAM levels were significantly lower in PCOS patients (50.01 ± 19.21 ng/ml) compared to controls (68.26 ± 29.29 ng/ml) (n = 42 in each group, *P* = 0.001; Fig 4C).

## Discussion

It is exceedingly difficult to create a model that reflects all phenotypes of PCOS because of the syndrome’s heterogeneity. In the present study, we demonstrated that prenatal androgenization of the ICR mouse with DHT can induce many characteristics of PCOS similar to those observed in previous studies using the C57/BL6 mouse [18–21]. The PNA mouse model exhibits disrupted estrous cyclicity, impaired fertility, elevated serum testosterone levels, and increased numbers of small antral follicles. Using this model, we have identified novel candidate genes for the hyperandrogenic phenotype of PCOS, including *MTR*. In the PNA mice and hyperandrogenic phenotype of women with PCOS, the expression of *MTR* was decreased and levels of its target, SAM, were also decreased in serum compared with the normal controls. MTR is crucial to one-carbon metabolism and the folate one-carbon pool pathway, while SAM is the main methyl donor of DNA methylation. Thus the present study indicates that the hyperandrogenic phenotype of PCOS is linked to dysregulated one-carbon metabolism and will likely affect DNA methylation. This may provide new clues in our understanding and treatment of the hyperandrogenic phenotype of PCOS.

Our study shows that the PNA mouse is an ideal model for emulating the hyperandrogenic phenotype of PCOS in women. Disrupted estrous cycles in PNA mice are mainly due to an absence of proestrus, increased duration of estrus and metestrus, and even a continuous switching between estrus and metestrus. Previous studies were controversial with respect to the extended duration of diestrus[18, 19], estrus [20], and metestrus[21]; but an absence of proestrus was representative of this phenotype. Disrupted estrous cyclicity indicates abnormal ovulation in PNA mice, and this is similar to the abnormal menstrual patterns seen in patients with PCOS. Fewer mouse litters and smaller litter sizes in the fertility test paralleled observations for human infertility in the hyperandrongenic phenotype of PCOS.

About 70%-90% of PCOS women with hyperandrogenism were defined either clinically or biochemically [22, 23]. Previous studies indicated that *in-utero* androgen exposure in humans can cause PCOS in adulthood[24], and PNA treatment in mice was used to mimic the process of *in-utero* androgen exposure. PNA mice showed increased androgen levels in adulthood rather than before puberty, indicating that hyperandrogenism in PCOS is due to altered function of the hypothalamic—pituitary—gonadal axis. LH hypersecretion is a common feature of PCOS, where LH is believed to enhance ovarian androgen production. Meanwhile, hyperandrogenism can further elevate LH level through diminishing negative feedback, forming a vicious circle[25]. Hyperandrongenism can also promote the development of PCOS, and affect hypothalamic—pituitary function, resulting in abnormal ovarian function and folliculogenesis. In the present study, genes involved in androgen biosynthesis and LH secretion, ovarian function, and folliculogenesis were found to be abnormally expressed using microarray analysis. For example, expression of *Nppc* was increased in PNA mice. CNP (C-type natriuretic peptide), encoded by *NPPC*, is downstream of FSH in the regulation of follicle development, and is secreted by granulosa cells of secondary and antral follicles to stimulate follicular development [26]. In addition, CNP is expressed in cumulus cells of antral and preovulatory follicles, so as to suppress oocyte maturation [27]. These results are consistent with increased antral follicle number and lack of mature follicles in PCOS. Chronic low-grade inflammation is also a common feature of PCOS according to previous studies [28], in light of which many genes encoding cytokines, especially chemokines, were abnormally increased in PNA mice, suggesting that the hyperandrogenic phenotype of PCOS is potentially associated with inflammation.

It appears to be paradoxical that overly activated follicles and infertility would coincide in PCOS, but a defect in follicle maturation is certainly a key factor. The reason for an increase in antral follicles leading to decreased follicular maturation in PCOS may be explained by nutritional factors. It is possible that nutrients are inadequate to support excessive antral follicle development and maturation. Previous studies have demonstrated that PCOS patients have altered plasma amino acid levels, including reduced Met level[29, 30]. Met plays an important role in the one-carbon metabolism pathway. SAM, the active form of Met, is the universal substrate for all protein and DNA methylation reactions, and is also involved in the synthesis of purines and pyrimidines. In this study, we found that *Bhmt* and *Mtr*, which encode enzymes that generate Met, were downregulated.

*BHMT* is an important gene in the one-carbon metabolism pathway, and its downregulation could lead to hyperhomocysteinemia[31]. BHMT was demonstrated to be upregulated in a previous microarray analysis for androgen receptor-deficient mice at 8 weeks of age [32].Consistently, the PNA mice in our study have decreased expression of *BHMT* and increased circulating testosterone.

In this study, we demonstrate for the first time that the expression of MTR is decreased in both PNA mice and women with the hyperandrogenic phenotype of PCOS. A previous study indicated that a single nucleotide polymorphism (SNP) was present in the *MTR* gene (*MTR* 2756 A>G)among south Asian women with similar frequency of minor alleles between women with and without PCOS[33]; Despite the small sample size (N = 21:9), the study interestingly showed that women in the control group had significantly lower hemoglobin and even manifested mild-to-moderate anemia (91, 85–101 vs. 123,105–129); the study, however, did not distinguish among different phenotype of PCOS [33]. In our study, we found that *MTR* expressionwas decreased in PNA mice starting from 3 weeks of age, whileT levels remained the same at 3weeks and was higher in PNA mice at 2 months of age. It appears that the inhibited *MTR* expression precedes increased circulating T, indicating a potential causal relationship between *MTR* and T levels. Nevertheless, further investigation is needed to confirm the causality.

MTR is known to play a key role in the folate one-carbon pool pathway and one-carbon metabolism pathway, and the main purpose of MTR is to regenerate Met in the SAM cycle; it also serves an essential function by allowing the SAM cycle to perpetuate without a constant influx of Met. The overall reaction converts 5-methyltetrahydrofolate into THF while transferring a methyl group to Hcy to form Met, while Met is further used to synthesize SAM under ATP action. MTR is the only mammalian enzyme that metabolizes N^5^-MeTHF to regenerate the active cofactor THF [15]. Therefore, a defect in MTR would lead to hyperhomocysteinemia, and low levels of Met and THF. Herein, we showed that serum SAM levels were significantly lower in PNA mice and the hyperandrogenic phenotype of women with PCOS. This suggests that down-regulation of *MTR* may reduce methionine levels and result in decreased SAM levels with PCOS.

Hcy is a non-essential amino acid formed by demethylation of Met, and previous studies showed that patients with PCOS exhibited elevated Hcy concentrations in their serum [34] and follicular fluid. Elevated Hcy levels in follicular fluid are now associated with poor-quality oocytes and embryos in PCOS patients undergoing assisted reproduction [35]. Some investigators have demonstrated that defective homocysteine re-methylation was found in MTR heterozygous knockout mice [36]. Our study showed that *MTR* was downregulated in GCs of PCOS patients, and that *Mtr* and *Bhmt* were downregulated in ovaries of PNA mice, revealing a reason for the elevated Hcy levels in PCOS patients.

Decreased SAM levels and hyperhomocysteinemia further indicates dysregulation of *MTR* with the hyperandrogenic phenotype of PCOS. Thus replenishing SAM or *MTR* could potentially benefit the hyperandrogenic phenotype of PCOS. Previous studies indicated that folate therapy reduced serum Hcy levels in patients with coronary artery disease [37], and the same phenomenon was observed in patients with PCOS [38]. It was also reported that folate supplementation in patients with PCOS had beneficial effects on metabolic profiles [39], and on inflammatory factors and biomarkers of oxidative stress [40].Furthermore, preconception folic acid supplementation was associated with suppression of the inflammatory pathway and upregulation of the HDL pathway in human follicular fluid[41]. It appears that folate may benefit patients with PCOS by compensating for the low expression of MTR.

Epigenetic mechanisms serve to elucidate the etiology of PCOS in the context of specific environmental factors before and after birth [24, 42].Studies have shown genome-wide DNA methylation difference between PCOS and normal ovaries [43]. In the present study, we demonstrated that levels of SAM, the main methyl donor of DNA methylation, were decreased with the hyperandrogenic phenotype of PCOS. This notion may explain the abnormal methylation patterns observed in PCOS.

It should be noted that because human ovarian tissue is extraordinarily difficult to obtain, we used GCs to validate the data generated from the PNA mouse. Results from this study should be interpreted with caution considering the relatively small number of samples, which could potentially introduce bias error. This study entailed an animal model and cells from women with the hyperandrogenic phenotype of PCOS; thus, whether these same results would be observed in PCOS women without hyperandrogenism would certainly require further investigation.

## Conclusions

Prenatal androgenization of the ICR mouse with DHT can replicate most of the common clinical features of PCOS, especially the hyperandrogenic phenotype of PCOS. By using this mouse model we demonstrate that PCOS is characterized by decreased *MTR* expression and serum SAM, which can potentially explain the dysregulated DNA methylation patterns in PCOS patients. Our evidence for the first time shows that an abnormal one-carbon metabolism pathway is linked to the hyperandrogenic phenotype of PCOS, the causality of which will be examined in our ongoing research.

## Supporting information

S1 Table Primer sequences used for qRT-PCR. (DOCX)

S2 Table Pathway enrichment of different expressed genes. (DOCX)

S3 Table Biological process of different expressed genes. (DOCX)

S4 Table Molecular function of different expressed genes. (DOCX)

S1 Fig Estrous cycle, fertility and follicles counting of mouse. (A) PNA mice exhibited prolonged estrous cycles. (B) Percent of days spent in each estrous cycle stage.(C) (D) PNA mice produced fewer litters and smaller litter sizes. (F) T levels in adult diestrus mice.(E) Body weight of mice. (F) Ovarian weight of mice. (G) Ovarian index of mice.(*: *P*< 0.05).
(TIF)

S2 Fig Sample quality control information. (A) Results summary table. (B) (C) Result of Agilent 2100 Bioanalyzer.
(TIF)

S3 Fig Different expressed MTR in 3 weeks and 3 months mice. (A) Expression of MTR in ovaries decreased in 3 months control mice. (B) Expression of MTR in ovaries did not change in PNA mice.
(TIF)
