# Supplementary material for: Attenuated expression of MTR in both prenatally androgenized mice and women with the hyperandrogenic phenotype of PCOS
Source: PLoS One. 2017 Dec 12;12(12):e0187427. doi: 10.1371/journal.pone.0187427 (PMC5726624; doi:10.1371/journal.pone.0187427)
Supplement: S1 Table — (DOCX) [file pone.0187427.s001.docx]

**Supporting Information**

**S1 Table. Primer sequences used for qRT-PCR.**

| **Gene** | **Sequence 5' - 3'** | **Size (bp)** |
| --- | --- | --- |
| *Aldh1a7* | F: GCAGCGTCCAGGCTTTTTG  R: CGGAGTTCAGAGGATTTCCTAGA | 103 |
| *Bhmt* | F: TTAGAACGCTTAAATGCCGGAG  R: GATGAAGCTGACGAACTGCCT | 142 |
| *Mtr* | F: TCCTCCTCGGCCTATCTTTATTT  R: GGTCCGAATGAGACACGCT | 107 |
| *Nrcam* | F: AGTGAGCGGGTTTCCCAAG  R: GCTGGATGGTTTGAGTGTGATT | 118 |
| *Ptprg* | F: TCCGCAGACGAAAGGCATC  R: GCTCCTGGTATTCATCGCCAA | 159 |
| *Mouse 18s* | F: ATGGCCGTTCTTAGTTGGTG  R: CGGACATCTAAGGGCATCAC | 183 |
| *MTR* | F: AGCGGGAGAAGCTAAACGAAG  R: CGGTAGGCCAAGTGTTCAAGG | 223 |
| *Human 18s* | F: CGGCTACCACATCCAAGGAA  R: CTGGAATTACCGCGGCT | 186 |
