# Supplementary material for: Attenuated expression of MTR in both prenatally androgenized mice and women with the hyperandrogenic phenotype of PCOS
Source: PLoS One. 2017 Dec 12;12(12):e0187427. doi: 10.1371/journal.pone.0187427 (PMC5726624; doi:10.1371/journal.pone.0187427)
Supplement: S2 Table — (DOCX) [file pone.0187427.s002.docx]

**S2 Table. Pathway enrichment of different expressed genes.**

| **Gene Set Name** | **No. of genes** | ***P* value** |
| --- | --- | --- |
| KEGG_PATHWAYS_IN_CANCER | 32 | 5.18E-12 |
| KEGG_CYTOKINE_CYTOKINE_RECEPTOR_INTERAERACTION | 25 | 4.96E-09 |
| KEGG_CHEMOKINE_SIGNALING_PATHWAY | 21 | 5.85E-09 |
| KEGG_WNT_SIGNALING_PATHWAY | 15 | 1.08E-05 |
| KEGG_MELANOGENESIS | 12 | 2.58E-05 |
| KEGG_MAPK_SIGNALING_PATHWAY | 19 | 2.73E-05 |
| KEGG_ERBB_SIGNALING_PATHWAY | 11 | 2.73E-05 |
| KEGG_BASAL_CELL_CARCINOMA | 9 | 2.84E-05 |
| KEGG_AXON_GUIDANCE | 13 | 2.86E-05 |
| BIOCARTA_BIOPEPTIDES_PATHWAY | 8 | 5.04E-05 |
