# Supplementary material for: Attenuated expression of MTR in both prenatally androgenized mice and women with the hyperandrogenic phenotype of PCOS
Source: PLoS One. 2017 Dec 12;12(12):e0187427. doi: 10.1371/journal.pone.0187427 (PMC5726624; doi:10.1371/journal.pone.0187427)
Supplement: S3 Table — (DOCX) [file pone.0187427.s003.docx]

**S3 Table. Biological process of different expressed genes.**

| **GO category** | **No. of genes** | ***P* value** |
| --- | --- | --- |
| SIGNAL_TRANSDUCTION | 115 | 3.04E-33 |
| MULTICELLULAR_ORGANISMAL_DEVELOPMENT | 83 | 5.54E-27 |
| ANATOMICAL_STRUCTURE_DEVELOPMENT | 81 | 9.07E-27 |
| SYSTEM_DEVELOPMENT | 69 | 9.86E-23 |
| BIOPOLYMER_METABOLIC_PROCESS | 92 | 6.06E-19 |
| CELLULAR_PROTEIN_METABOLIC_PROCESS | 70 | 2.89E-17 |
| CELLULAR_MACROMOLECULE_METABOLIC_PROCEOCESS | 70 | 4.82E-17 |
| PROTEIN_METABOLIC_PROCESS | 73 | 7.43E-17 |
| CELL_DEVELOPMENT | 46 | 5.06E-15 |
| RESPONSE_TO_EXTERNAL_STIMULUS | 34 | 5.54E-15 |
