# Supplementary material for: Attenuated expression of MTR in both prenatally androgenized mice and women with the hyperandrogenic phenotype of PCOS
Source: PLoS One. 2017 Dec 12;12(12):e0187427. doi: 10.1371/journal.pone.0187427 (PMC5726624; doi:10.1371/journal.pone.0187427)
Supplement: S4 Table — (DOCX) [file pone.0187427.s004.docx]

**S4 Table. Molecular function of different expressed genes.**

| **GO category** | **No. of genes** | ***P* value** |
| --- | --- | --- |
| RECEPTOR_BINDING | 41 | 1.79E-17 |
| RECEPTOR_ACTIVITY | 42 | 6.86E-12 |
| ENZYME_REGULATOR_ACTIVITY | 31 | 6.86E-12 |
| G_PROTEIN_COUPLED_RECEPTOR_BINDING | 12 | 1.76E-08 |
| TRANSMEMBRANE_RECEPTOR_ACTIVITY | 28 | 2.90E-07 |
| CYTOSKELETAL_PROTEIN_BINDING | 16 | 2.24E-06 |
| CHEMOKINE_ACTIVITY | 9 | 2.75E-06 |
| CHEMOKINE_RECEPTOR_BINDING | 9 | 2.78E-06 |
| ENZYME_ACTIVATOR_ACTIVITY | 14 | 2.78E-06 |
| PROTEIN_KINASE_REGULATOR_ACTIVITY | 8 | 1.55E-05 |
