# Supplementary figures and images for: Attenuated expression of MTR in both prenatally androgenized mice and women with the hyperandrogenic phenotype of PCOS
Source: PLoS One. 2017 Dec 12;12(12):e0187427. doi: 10.1371/journal.pone.0187427 (PMC5726624; doi:10.1371/journal.pone.0187427)

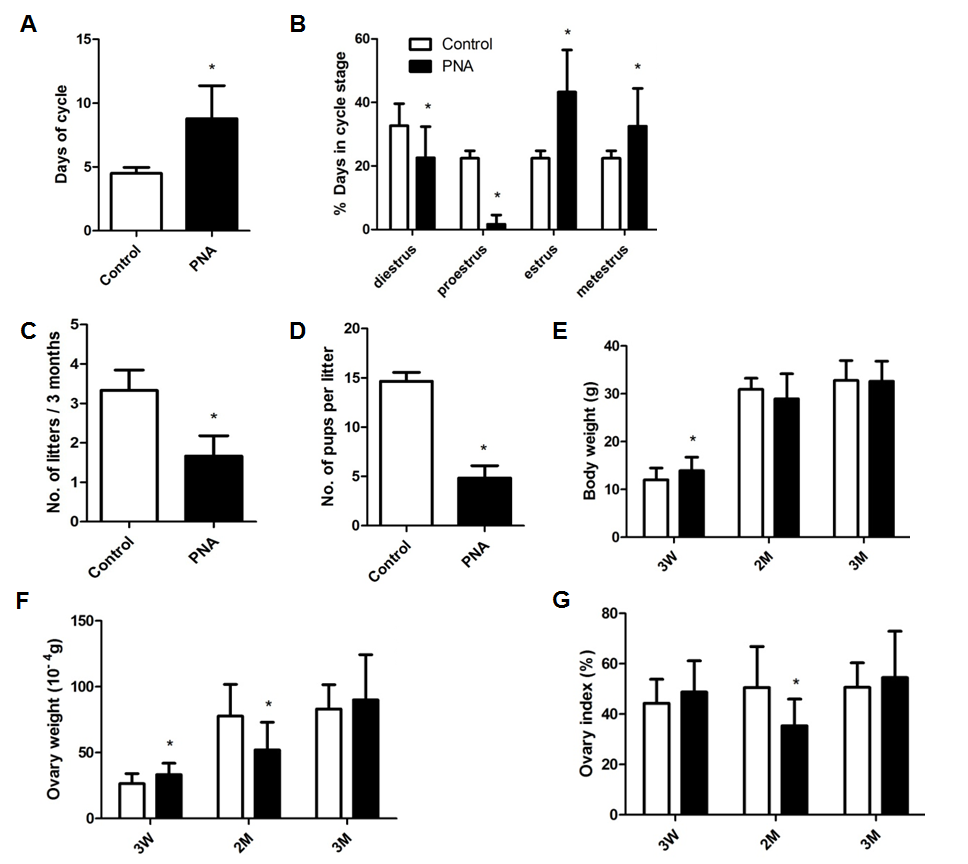

Supplement: S1 Fig — (A) PNA mice exhibited prolonged estrous cycles. (B) Percent of days spent in each estrous cycle stage.(C) (D) PNA mice produced fewer litters and smaller litter sizes. (F) T levels in adult diestrus mice.(E) Body weight of mice. (F) Ovarian weight of mice. (G) Ovarian index of mice.(*: P< 0.05). (TIF) [file pone.0187427.s005.tif]

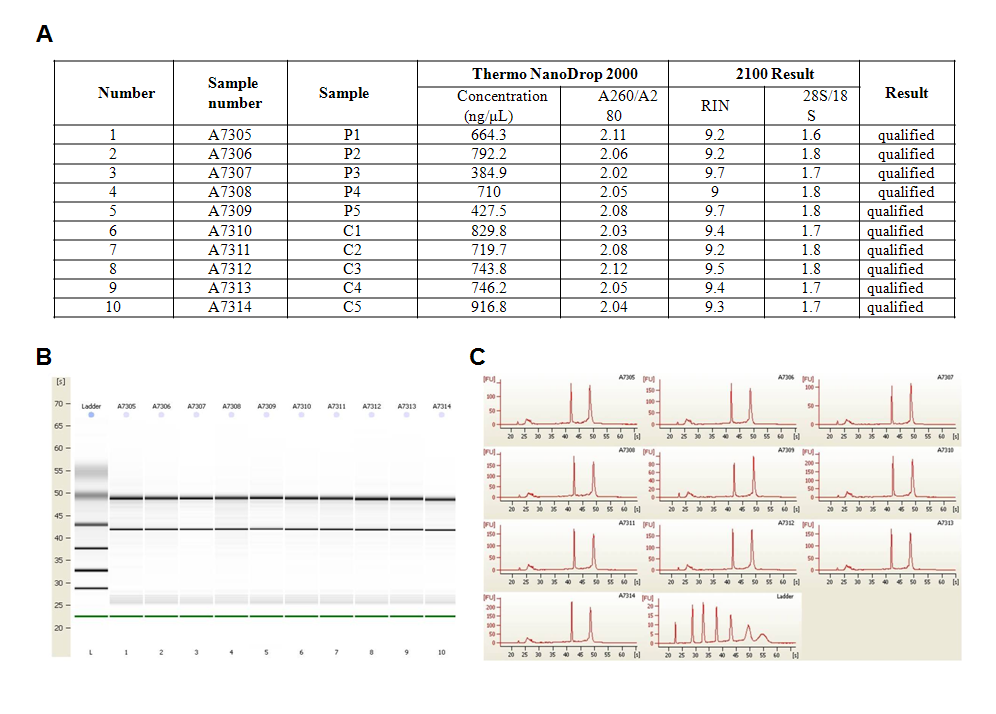

Supplement: S2 Fig — (A) Results summary table. (B) (C) Result of Agilent 2100 Bioanalyzer. (TIF) [file pone.0187427.s006.tif]

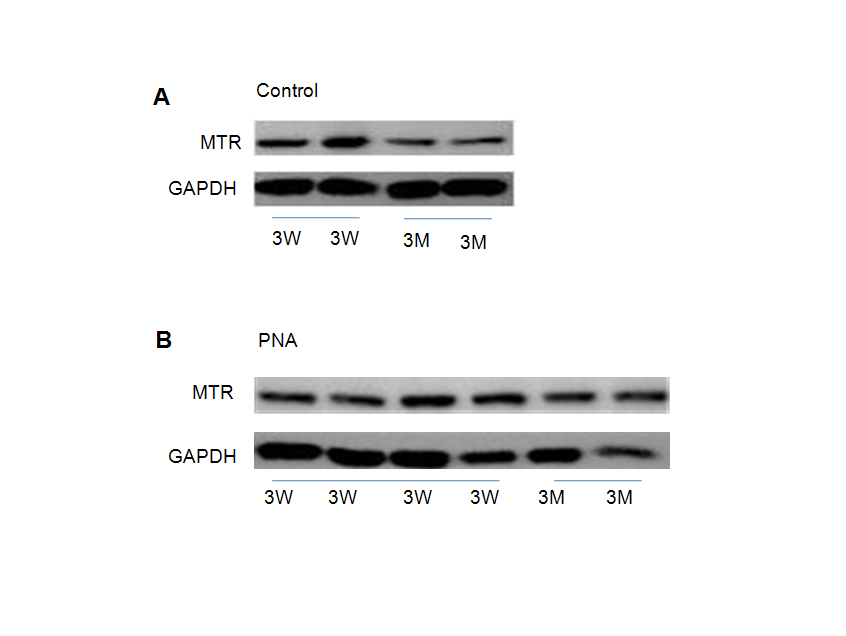

Supplement: S3 Fig — (A) Expression of MTR in ovaries decreased in 3 months control mice. (B) Expression of MTR in ovaries did not change in PNA mice. (TIF) [file pone.0187427.s007.tif]
